# Cellular Metabolites Regulate Central Nucleic Acid Sensing Pathways

**DOI:** 10.3389/fimmu.2021.635738

**Published:** 2021-02-19

**Authors:** Julia Blay-Cadanet, Alice Pedersen, Christian Kanstrup Holm

**Affiliations:** Department of Biomedicin, Aarhus University, Aarhus, Denmark

**Keywords:** metabolites, lactate, succinate, itaconate, MAVS, STING, toll-like receptors

## Abstract

Detection of pathogen-derived DNA or RNA species by cellular nucleic acid sensors prompts release of anti-microbial interferons and cytokines. In contrast to their protective anti-microbial functions, inappropriate or excessive activation of nucleic acid sensors can cause inflammatory diseases. Nucleic acid sensing is therefore tightly controlled by regulatory factors acting through both transcriptional and post-transcriptional mechanisms. Recently, it has become clearer that metabolic pathways—previously thought to be unconnected with immune responses—can influence nucleic acid sensing. This regulation can be observed when immune system cells undergo metabolic reprogramming in response to stimulation with pathogen-associated molecular patterns such as lipopolysaccharide from gram negative bacteria. Metabolic reprogramming leads to accumulation and secretion of metabolites, which have been mostly viewed as end-products of processes providing cellular energy and building blocks. However, metabolites have now been identified as important regulators of nucleic acid sensing. This mini-review aims to outline current knowledge on regulation of central nucleic acid sensing pathways by metabolites during metabolic reprogramming.

## Introduction

**Figure 1:**
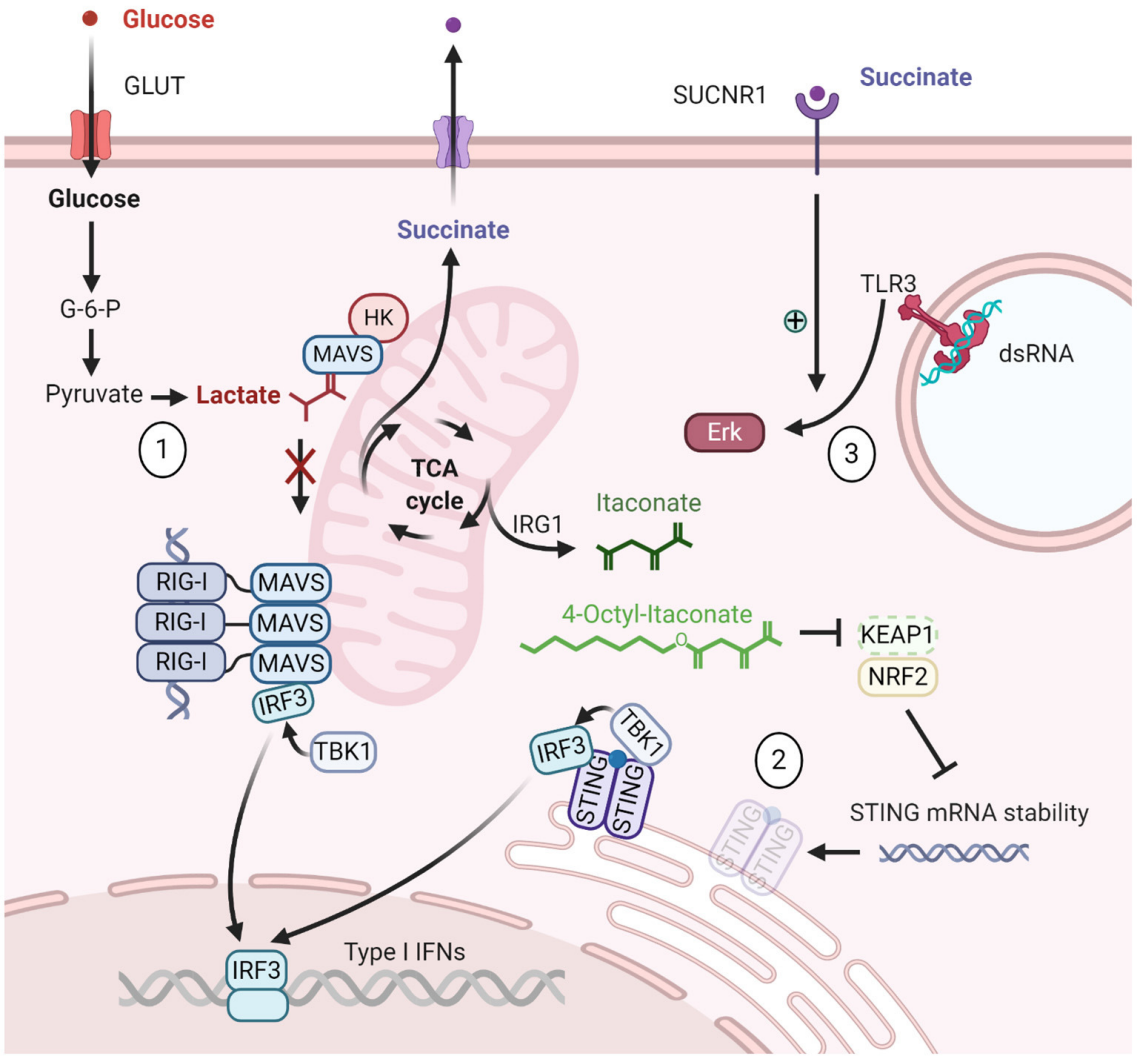
Overview of metabolites regulating nucleic acid sensing pathways. (1) Lactate inhibits the RIG-I-MAVS pathway. (2) 4-Octyl-itaconate inhibits the stability of the STING mRNA. (3) Succinate enhances Erk signaling through TLR3. Created with BioRender.com.

The innate immune response employs a range of pattern recognition receptors (PRRs) to detect danger-associated molecular patterns and conserved microbial pathogen-associated molecular patterns (PAMPs) to trigger defense mechanisms (1). Nucleic acids derived from infectious pathogens are central PAMPs that can be detected by a selection of PRRs—the nucleic acid sensors. Nucleic acid sensing occurs through membrane-associated receptors of the Toll-like receptor (TLR) family and through the sensors of cytosolic nucleic acids RIG-I-like receptors (RLRs), cyclic GMP-AMP synthase (cGAS), and absent in melanoma 2. Ligation of these sensors induces expression of both type I interferons (IFNs) and pro-inflammatory mediators. Although the nucleic acid sensors are critical for protection against infection, they can also be the cause of inflammatory diseases (2). These sensors and their downstream effectors are thus subject to important regulatory mechanisms aimed at preventing excessive pro-inflammatory signals. Regulation has already been described to target nucleic acid sensing through proteasomal degradation of central signaling components, through pre- and post-transcriptional regulation of expression and through enzymatic degradation of the activating nucleic acid agonists (3). Intriguingly, a novel class of underappreciated regulators of nucleic acid sensing has recently emerged (Figure 1). These regulators are metabolites derived from metabolic pathways and processes providing energy and building blocks for basic cellular processes. In particular, these metabolites accumulate during a process now known as metabolic reprogramming.

Metabolic reprogramming as a phenomenon was first identified in cancer cells, which display increased consumption of glucose and release of lactate—also known as the Warburg effect. It is now clear that metabolic reprogramming also occurs in other cells. For example, immune cells such as macrophages and dendritic cells (DCs) undergo metabolic reprograming upon stimulation with bacteria-derived ligand lipopolysaccharide (LPS) and ligands mimicking microbial RNA. (4–7). There is evidence that DNA also induces metabolic reprogramming of immune cells. For instance, oxidized DNA from extracellular vesicles formed by the *T. cruzi* parasite causes proinflammatory reprogramming of macrophages (8). Furthermore, TLR9 mediates activation and metabolic reprogramming of plasmacytoid DCs toward glycolysis which is important for their type I IFN production (9).

Metabolic reprogramming is characterized by an elevated consumption of glucose, a disruption of the tricarboxylic acid (TCA) cycle and increased formation of a series of metabolites—most notably lactate, succinate, and itaconate (4–7, 10). Lactate is predominantly formed from pyruvate through the enzymatic control of lactate dehydrogenase (LDHA) (11). Although lactate levels in serum are used as a prognostic tool in many critical medical conditions (10), its potential to affect immune responses has largely been ignored. Intriguingly, studies now highlight lactate as an important cellular regulator of innate and adaptive immunity. Lactate has been shown to support cancer cell proliferation acting as an alternative source to feed the mitochondrial oxidative phosphorylation for efficient ATP production, it can also be metabolized into lipids, or even metabolized by the mitochondrial lactate dehydrogenase B for respiration (12). Also, lactate accumulating in melanomas suppresses T and natural killer (NK) cells function and survival, and thus enables tumors to escape T and NK cell-mediated tumor surveillance (13). Furthermore, tumor-derived lactate promotes tumor growth and M2-directed polarization of tumor-associated macrophages (14). This turns the tumor microenvironment into a more immunosuppressive state and thus enables tumors to evade immune responses.

Succinate is synthesized within the mitochondrial matrix as part of the TCA-cycle. Succinate links the TCA-cycle with the electron transport chain as succinate dehydrogenase (SDH, a.k.a. succinate-ubiquinone oxidoreductase or Complex II) transfers an electron from succinate to ubiquinone generating fumarate from succinate in the process. Succinate accumulates in LPS-stimulated macrophages (5, 15), but the effects of increased intracellular succinate levels on inflammatory responses remain unclear. While the succinate derivative dimethyl-succinate seems to increase relative expression of the pro-inflammatory cytokine interleukin 1β (IL-1β) (5), the cellular accumulation of endogenous succinate seems to occur after and not before LPS-induced IL-1β (15). Similarly, in experiments by Harber et al., dimethyl-succinate was found to trigger anti-inflammatory responses through a mechanism operating independently of its receptor succinate receptor 1 (SUCNR1) (16). By contrast, unmodified succinate secreted into the extracellular space is now demonstrated to activate pro-inflammatory pathways through the cell surface SUCNR1 which is highly expressed in macrophages (17) and DCs (16). Therefore, it is still unclear to what extend the pro- or anti-inflammatory effects of unmodified endogenous succinate overlap with those demonstrated for modified variants of succinate. Like other metabolites, succinate also accumulates in cancer cells. This accumulation favors cancer progression since it induces epigenetic alterations, alters cancer cell metabolism, promotes epithelial-to mesenchymal transition, migration and invasion, and promotes angiogenesis (18).

Itaconate, is a TCA-cycle-derived metabolite found to accumulate in several models of inflammation including Mycobacterium tuberculosis-infection (19) and LPS-treated macrophages (20). Itaconate is formed from enzymatic conversion of cis-aconitate by the immune responsive gene 1 (IRG1) (21). Expression levels of IRG1 and of itaconate are low to absent in resting macrophages but are highly induced through TLR stimulation with LPS (22). Lampropoulou et al. (20), then described how addition of the itaconate derivative dimethyl-itaconate (DI) inhibited infection-induced inflammatory cytokines and affected macrophage differentiation/activation by downregulating proinflammatory transcripts and inhibiting the inflammasome.

Many advances have been made in the past few years toward recognizing the importance of altered metabolism in health and disease. Metabolic changes affecting multiple cells in pathologies, such as obesity and cancer, confirm that there is a surging focus on improving our understanding of the interphase of metabolism and innate immune responses. Obesity is characterized by metabolic stress due to elevated levels of free fatty acids. In this condition, palmitic acid can provoke leakage of mitochondrial DNA into the cytosol. This activates the cGAS-STING pathway to induce the expression of intercellular adhesion molecule 1 and monocyte-endothelial cell adhesion, which results in endothelial inflammation (23). The link between metabolic changes in obesity and nucleic acid sensing underlines the importance of regulation of nucleic acid sensing by metabolites. Therefore, this mini-review outlines novel discoveries on how cellular metabolites influence nucleic acid sensing.

## Nucleic Acids Sensors

At steady state, positive and negative regulators secure an appropriate balance between immune activation and suppression. The mechanisms that control nucleic acid sensing include regulation of ligand availability and posttranslational modifications of both the nucleic acid sensors and the signaling adaptor proteins (3). Traditionally, metabolites derived from cellular pathways such as glycolysis and the TCA-cycle were viewed solely as end-products of cellular processes. Interestingly, there is now evidence that metabolites can affect nucleic acid sensing pathways as well and alter the immune response.

### TLRs

In the year 2000, the discovery that TLR9 binds and detects DNA marked the first report of nucleic acid sensing by innate cellular PRRs (24). Quickly thereafter, several other nucleic acid sensors were identified among the TLR family members including, TLR3 (25), 7 and 8 (26) which detect pathogen-derived RNA in endosomes. TLR9 and TLR7/8 signal through myeloid differentiation primary response 88 (MyD88) whereas TLR3 signals through TIR-domain-containing adaptor-inducing interferon-b (TRIF) to induce type I IFNs and pro-inflammatory cytokines (2).

As mentioned above, itaconate is strongly induced upon macrophage activation through TLR stimulation. Subsequently, several studies reported strong anti-inflammatory effects of cell permeable derivatives of itaconate including dimethyl-itaconate (DI) and 4-Octyl-itaconate (4-OI) (20, 27). At least to some extent, these effects have been confirmed using unmodified itaconate (15). Mills et al. (27), observed that upon TLR signaling, itaconate is also able to downregulate IL-1β formation through activation of the kelch-like ECH-associated protein 1/nuclear factor erythroid 2-related factor 2 (KEAP1/NRF2) anti-oxidant response.

It has also been observed that immune responses upon TLR signaling can be enhanced by interaction of succinate with its receptor SUCNR1. It was previously observed by He et al. (28), that succinate can activate extracellular signal-regulated kinase (Erk), a downstream component of the TLR pathways. Therefore, Rubic et al. (16), investigated whether succinate had an effect directly on the TLRs. They observed that Erk1/2 phosphorylation was slightly induced by the TLR3 stimulator poly I:C alone, but enhanced when succinate was simultaneously present. Consequently, they reported that in combination with some nucleic acid analogs like poly I:C (TLR3 agonist) or imiquimod (TLR7 agonist), succinate potentiates production and secretion (by direct post-transcriptional effect) of pro-inflammatory mediators such as IL-1β and TNFα.

### RIG-I—MAVS

The RNA sensors RIG-I and melanoma differentiation-associated protein 5 (MDA5) detect double stranded (ds) RNA to initiate an antiviral response through the signaling adaptor mitochondrial antiviral signaling (MAVS) (29–34). RIG-I is kept in a closed confirmation in absence of RNA. However, upon dsRNA binding, a structural change occurs that enables RIG-I signaling through MAVS (3). The IκB kinase β (IKKβ) or TANK-binding kinase 1 (TBK1) phosphorylate MAVS and this enables IFN-regulatory factor 3 (IRF3) recruitment. TBK1 phosphorylates the recruited IRF3—and phosphorylated IRF3 homodimerizes and translocates to the nucleus where it induces type I IFN expression (35). Apart from IRF3, MAVS activation also leads to phosphorylation of IκBα (33) and release of nuclear factor κB (NF-κB) that will translocate to the nucleus and mediate expression of proinflammatory genes.

A recent study established a link between this nucleic acid sensing pathway and cellular metabolism. Zhang et al. (36), noted a decrease in the levels of distinct metabolites associated with the glycolytic process—phosphoenolpyruvate, pyruvate, and lactate—during RLR-stimulation, indicating that upon RLR-activation, glucose metabolism is inhibited.

The underlying mechanism is based on the exclusion of hexokinase 2 (HK2) from the mitochondria upon RLR activation. HK2 mediates the conversion of glucose to glucose-6-phosphate and its inhibition thus prevents the first step in glycolysis. In this manner, RLR activation inhibits glycolytic flux through inhibitory displacement of HK2 from the mitochondria. The authors next asked why prevention of glycolysis was necessary for optimal induction of type I IFN by RIG-I. Here, they discovered that lactate, the end product of anaerobic glycolysis, inhibited RLR signaling. Thus, inhibition of glycolysis was necessary to prevent the inhibitory effect of lactate. Co-precipitation experiments and domain mapping showed that lactate directly interacted with the transmembrane domain of MAVS and that this modification of MAVS inhibited its polymerization and subsequently the downstream induction of type I IFNs. Thus, lactate is a metabolite that inhibits RLR-signaling, hereby connecting anaerobic glycolysis to nucleic acid sensing (36).

There is also evidence that lactate suppresses RLR-signaling in a more physiologically relevant context *in vivo*. Using the Hepatitis B virus (HBV) hydrodynamic injection (HI) mouse model, Zhou et al. (37), observed an increase in pyruvate and lactate production by HBV *in vivo*. By administrating sodium oxamate with the aim to reduce lactate production, they measured decreased viral-DNA levels in serum and reduced HBV-replication intermediates in mouse liver tissues. Accordingly, increasing lactate levels by administration of sodium lactate resulted in an opposite effect. In addition, administration of sodium lactate in mice reduced production of IFN-b. By testing the HBV HI mouse model in WT/*Ifnar*^+/+^ and *Ifnar1*^−/−^ mice, they demonstrated that HBV evades the immune response by suppressing RLR-mediated IFN production in a manner dependent on LDHA-mediated lactate production (37). This could be an important mechanism for the regulation of anti-viral responses and an obvious target of human pathogenic viruses to prevent optimal induction of type I IFNs, also a mechanism that could be utilized by cancer cells to avoid IFN-stimulation of immune cells.

### cGAS-STING

In 2006, Ishii and colleagues reported that transfection of dsDNA into living cells induced an antiviral response, that required TBK1, but was independent of TLRs. They furthermore observed that the response to dsDNA yielded protection against both DNA and RNA viruses (38). It is now known that cGAS is a central sensor of cytosolic DNA that relays its signal to the signaling adaptor protein stimulator of interferon genes (STING) through the generation of the second messenger cyclic GMP-AMP (cGAMP) (39, 40). Upon cGAMP-binding, STING dimers undergoes major structural changes that allow it to stack side-by-side into tetramer and higher-order oligomer structures—a process that depends on palmitoylation of specific STING residues (41–45). These changes allow TBK1 dimers to activate each other by autophosphorylation (46, 47). Activated TBK1 phosphorylates STING and phosphorylated STING serves as a docking site for IRF3. TBK1, in turn, phosphorylates IRF3. This phosphorylation enables IRF3 to homodimerize and translocate to the nucleus where it activates the transcription of type I IFNs (35).

The cGAS-STING pathway has recently been demonstrated to be suppressed during metabolic reprogramming. This suppression was mediated through decreased expression of STING itself (48). More specifically, activation of the transcription factor NRF2 during metabolic reprogramming suppresses STING expression through a mechanism that could depend on mRNA stability. The NRF2-inhibitor protein, KEAP1, targets NRF2 for proteasomal degradation at steady state conditions. However, several stimuli inactivate KEAP1 allowing NRF2 to translocate to the nucleus. This induces the transcription of NRF2 target genes that protect cells from death and regulate inflammatory responses. Olagnier et al. (48), showed that upon metabolic reprogramming by TLR4/7-activation and in response to the itaconate derivative 4-OI, NRF2 suppresses STING expression. Thus, cellular metabolites that induce NRF2 activation can in this manner indirectly target nucleic acid sensing through decreasing the expression of STING.

**Figure 2:**
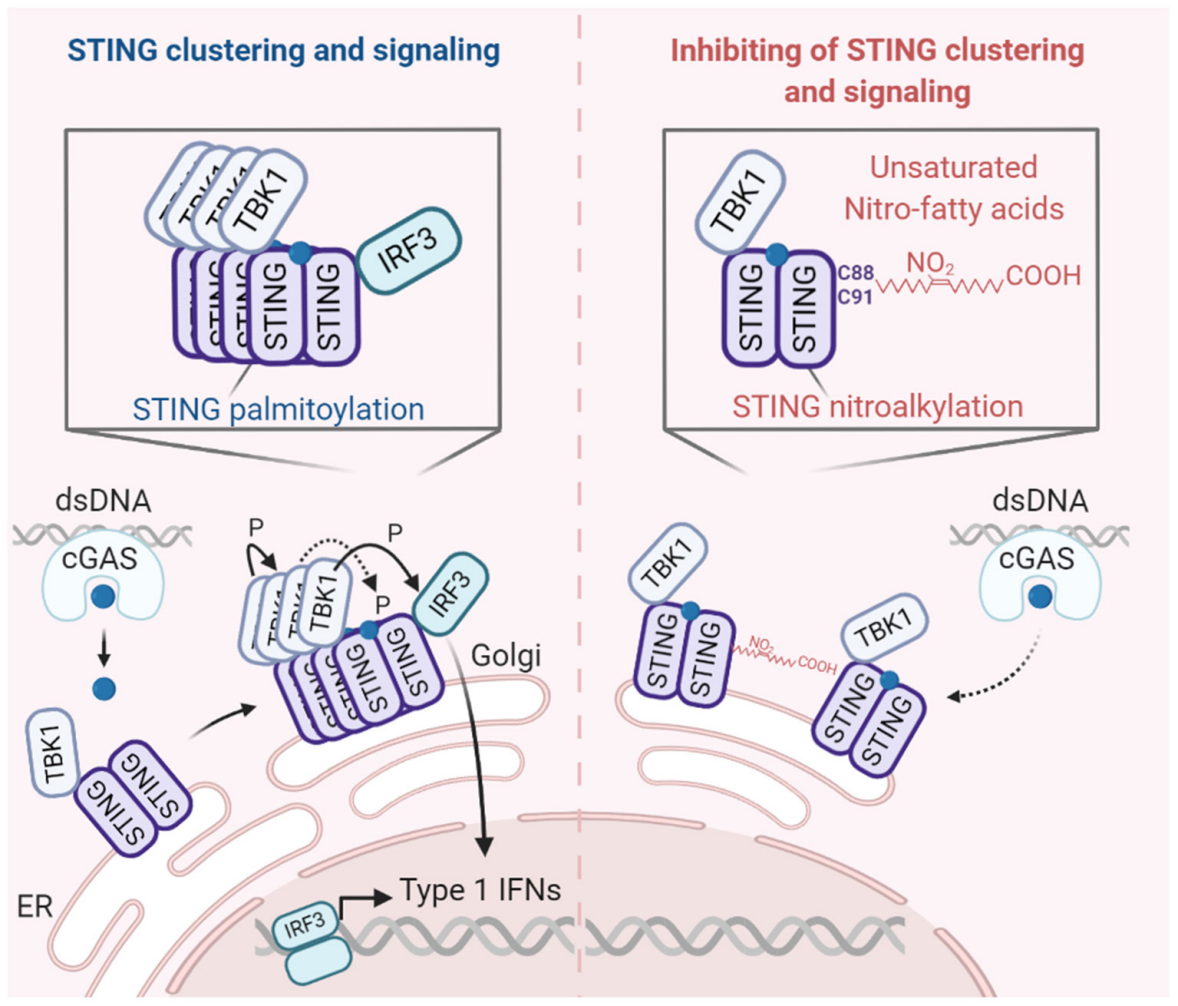
Nitro-fatty acids inhibit STING palmitoylation and signaling. Upon detection of dsDNA, cGAS catalyzes the formation of 2′3-cGAMP. STING resides in the ER as a dimer bound to TBK1. Upon cGAMP-binding, the STING dimers change conformations and stack side-by-side to form oligomers. TBK1 dimers, in turn, autophosphorylate and activate each other. Activated TBK1 phosphorylates adjacent STING dimers. Phosphorylated STING in turn serves as a docking site for IRF3. Upon phosphorylation by TBK1, phosphorylated IRF3 monomers disassociate from STING and homodimerize. The homodimer works as a transcription factor to induce type I IFNs. Palmitoylation of STING in the Golgi is important for STING clustering and signaling. Importantly, nitro-fatty acids inhibit STING palmitoylation and signaling. Created with BioRender.com.

Nitro-fatty acids are another group of metabolic by-products that are formed during infection and target the cGAS-STING signaling pathway (Figure 2). Nitro-fatty acids are electrophilic elements that react with nucleophilic donors such as cysteines and thiols (49, 50). Mechanistically, the nitro-fatty acids modify STING post-translationally through nitro-alkylation at cysteines 88 and 91. This modification prevents palmitoylation of STING, that is necessary for optimal STING activity, and thus inhibits it signaling. Interestingly, nitro-fatty acids inhibited release of type I IFN in cells from patients with STING-associated vasculopathy with onset in infancy (SAVI)—a genetic disorder caused by gain-of-function mutations in STING (51, 52). Thus, these metabolic by-products could be important regulators of nucleic acid sensing and possibly be utilized as therapeutics in STING-dependent inflammatory conditions.

## Implications and Conclusions

Our current understanding of immunity to infection is dominated by aggressive and highly inflammatory processes. Although these processes and pathways are effective in eliminating pathogens—they are also highly destructive and result in high degrees of collateral damage to otherwise healthy tissues. Recent advances suggest that basal cellular metabolic processes play a so far underappreciated role in the regulation of such damaging responses. Although great advances have been made in this field over the last handful of year, we have still only begun to elucidate the regulatory interphase of cellular metabolism and immunity. Metabolites that were previously thought to not affect immunity are now emerging as important regulators of both innate and adaptive immune responses.

Since many pathologies, such as obesity and cancer, carry metabolic alterations, there is a huge treatment potential in reshaping the metabolism in such conditions. For example, Pankowicz et al. (53) were able to treat hereditary tyrosinemia in mice, a metabolic liver disorder, by targeting the metabolic enzyme hydroxyphenylpyruvate dioxygenase rather than the disease-causing gene. This successfully demonstrates the usefulness of regulating changes in metabolism that could be an effective treatment strategy in other diseases.

In line with this, we have presented studies that identify metabolites as important regulators of nucleic acid sensing pathways pointing to a therapeutic potential of metabolites in inflammation. For instance, in the context of viral infections, it could have a great potential to target virus-induced production of lactate that inhibits the antiviral RLR-MAVS-IFN axis. Many viruses, such as SARS-CoV2 and influenza, cause disease by inducing general inflammation. In these cases, it could be fruitful to limit the disease-causing virus-induced inflammation by targeting nucleic acid sensing with metabolites that dampen the inflammation. Likewise, in inflammatory conditions, it could be beneficial to reduce inflammation by blocking STING signaling using for example nitro-fatty acids.

There is evidence indicating a cross talk between different pattern recognition pathways. For instance, LPS-induced inflammation and metabolic reprogramming through TLR4 lead to accumulation of metabolites, including lactate. Lactate, in turn, inhibits the RLR-MAVS pathway, which results in an anti-inflammatory effect. Such cross talk is important to keep in mind when we aim to modulate immune responses using metabolites.

The future is bound to reveal many more connections between metabolism and nucleic acid sensing that will greatly advance our understanding of how immune responses are kept in check. It is likely that these efforts will also lead to the identification of metabolites and metabolic pathways that can be targeted to either promote or suppress immune responses depending on the context and whether the goal is to increase immunity to cancer and infection or prevent this in the context of inflammatory diseases.

## Author Contributions

JB-C, AP, and CH drafted and finalized the manuscript. All authors contributed to the article and approved the submitted version.

## Conflict of Interest

The authors declare that the research was conducted in the absence of any commercial or financial relationships that could be construed as a potential conflict of interest.
